# It Takes Time to Be Cool: On the Relationship between Hyperthermia and Body Cooling in a Migrating Seaduck

**DOI:** 10.3389/fphys.2017.00532

**Published:** 2017-07-25

**Authors:** Magella Guillemette, Elias T. Polymeropoulos, Steven J. Portugal, David Pelletier

**Affiliations:** ^1^Departement de Biologie, Universite du Quebec a Rimouski Rimouski, QC, Canada; ^2^Institute for Marine and Antarctic Studies, University of Tasmania Hobart, TAS, Australia; ^3^School of Biological Sciences, Royal Holloway University of London Egham, United Kingdom; ^4^Departement de Biologie, Cegep de Rimouski Rimouski, QC, Canada

**Keywords:** body cooling, common eiders, endothermy, hyperthermia, migration, thermoregulation

## Abstract

The large amount of energy expended during flapping flight is associated with heat generated through the increased work of the flight muscles. This increased muscle work rate can manifest itself in core body temperature (T_b_) increase of 1–2°C in birds during flight. Therefore, episodic body cooling may be mandatory in migratory birds. To elucidate the thermoregulatory strategy of a short-distance migrant, common eiders (*Somateria mollissima*), we implanted data loggers in the body cavity of wild birds for 1 year, and report information on T_b_ during their entire migration for 19 individuals. We show that the mean body temperature during flight (T_bMean_) in the eiders was associated with rises in T_b_ ranging from 0.2 to 1.5°C, largely depending on flight duration. To understand how eiders are dealing with hyperthermia during migration, we first compare, at a daily scale, how T_b_ differs during migration using a before-after approach. Only a slight difference was found (0.05°C) between the after (40.30°C), the before (40.41°C) and the migration (40.36°C) periods, indicating that hyperthermia during flight had minimal impact at this time scale. Analyses at the scale of a flight cycle (flight plus stops on the water), however, clearly shows that eiders were closely regulating T_b_ during migration, as the relationship between the storage of heat during flight was highly correlated (slope = 1) with the level of heat dumping during stops, at both inter-individual and intra-individual levels. Because T_b_ at the start of a flight (T_bStart_) was significantly and positively related to T_b_ at the end of a flight (T_bEnd_), and the maximal attained T_b_ during a flight (T_bMax_), we conclude that in absence of sufficient body cooling during stopovers, eiders are likely to become increasingly hyperthermic during migration. Finally, we quantified the time spent cooling down during migration to be 36% of their daily (24 h) time budget, and conclude that behavioral body cooling in relation to hyperthermia represents an important time cost.

## Introduction

Homeothermic endotherms—animals that maintain a constant, regulated, body temperature—have the unique ability to maintain a high core body temperature (T_b_) across a wide range of ambient temperatures (T_a_; Crompton et al., 1978). Birds display the highest T_b_ of all endothermic animals, exceeding that of mammals, for example, on average by up to ≈2.5°C (Prinzinger et al., 1991). It has been proposed that such a high T_b_ in birds is prevalent to enhance the performance of powered flight through Q_10_—the factor by which the rate of a reaction increases for every 10 degree rise in temperature—driven effects on muscle contractile activity (Torre-Bueno, 1976). Although, birds are known to regulate body temperature to a high degree and within very narrow margins, there is building evidence that substantial deviations from normothermia naturally occur (Guillemette et al., 2016). Indeed, hypothermic events such as torpor is evident in many families of afrotropical birds while there are numerous studies reporting cases of controlled hypothermia as a strategy to conserve energy in temperate and arctic environments as well (McKechnie and Mzilikazi, 2011; Lewden et al., 2014). While T_b_ may be lowered as an energy-saving mechanism in many bird species, the result of the high-energy demands imposed by flight, with flapping flight often being described as the costliest locomotion mode among vertebrates (Butler and Woakes, 1990; Butler, 2016), is associated with the highest levels of known heat production among endotherms (Clarke and Rothery, 2008). It is estimated that 80–96% of the metabolic energy used during flapping flight in birds and bats is dissipated as heat, while only 4–20% is converted into mechanical power (Carpenter, 1986; Speakman and Thomas, 2003). Such dramatic increases in T_b_ during flight (Platania et al., 1986) require accurate regulation to avoid critical levels of hyperthermia being reached, and the associated deleterious physiological consequences such as excessive evaporative water loss (Boyles et al., 2011). Long-distance avian migrations can include non-stop flights of over 10,000 km, involving millions of wingbeats (Gill et al., 2005). Therefore, in flying birds, mechanisms for body cooling are likely to be an important feature of migration strategies, when flight time is considerably increased and the risk of hyperthermia may be highest.

To cope with the increases in T_b_ during long-duration flights, a diverse range of behavioral and physiological strategies have evolved in birds as a means to maintain a constant T_b_, and thus retain cellular homeostasis. Unlike endothermic mammals, however, birds lack sweat glands and thus rely on other mechanisms of effective heat loss. The respiratory tract, through panting or gular fluttering, plays an important role in evaporative heat loss in some species, while the plumage, skin, bill as well as the legs and feet are known important sites of heat exchange that may assist in alleviating the large water loss through the respiratory tract (Steen and Steen, 1965; Dawson, 1982; Giladi and Pinshow, 1999; Tattersall et al., 2016). Evaporative heat loss through the limbs has been well documented, notably in seabirds (Steen and Steen, 1965; Baudinette et al., 1976). The arteries and veins in the legs of many birds lie in close contact with each other, forming a rete mirabile (a net-like-complex of arteries and veins), and function as a countercurrent heat exchange system (Midtgård, 2008). In addition, many birds can adjust the blood flow through the legs by muscular contraction, and can thus regulate heat loss to some degree depending on environmental conditions (Kilgore and Schmidt-Nielsen, 1975).

The behavioral and physiological mechanisms employed by many birds to regulate body temperature are most pertinent during long-duration flights, chiefly those associated with migration when heat loss becomes a particularly challenging problem. Some species may increase their flight altitude to reach cooler temperatures or wind conditions (Torre-Bueno, 1976; Bäckman and Alerstam, 2001) whereas other species are known to fly predominantly at night-time in cooler temperatures which may be related to thermoregulation (Kerlinger and Moore, 1989). For many migratory birds, stopovers are a crucial component of migration and serve to replenish and restore energy reserves (Moore and Kerlinger, 1987), but such breaks from flight may also play a role in avoiding hyperthermic conditions and maintaining homeostasis.

Recently, Guillemette et al. (2016) demonstrated that common eiders (*Somateria mollissima*) experience large departures from normothermia during migratory flights. These increases in T_b_ ranged on average between 0.2 and 2.4°C during migratory flights, and were likely due to the high wing-loading found in eiders (a large body to small wings ratio), the morphology of which is linked to their diving lifestyle. Indirect evidence that eiders proactively avoid deleterious levels of hyperthermia during flight is given by their migratory style, whereby they use a stop-and-go strategy where they perform many flights of short duration (15 min) during which T_b_ increased by 1°C on average (Guillemette et al., 2016). In addition, about 60% of the migratory flights were stopped while T_b_ was still increasing (Guillemette et al., 2016). Therefore, it appears that behaviorally mediated body cooling, might be an important component of the thermoregulatory strategy during migration for eiders.

In this paper, we evaluate the role of body cooling during migration stop-overs of common eiders while they migrate from breeding grounds to their late-summer molting sites. More specifically, we (1) compare daily T_b_ during migration to T_b_ prior to the migratory flights, and predict that daily T_b_ during migration is higher overall than daily T_b_ during the pre-migratory phase when flight time is significantly lower. We (2) predict that hyperthermia is a constraint to flight duration in migrating eiders, and therefore they should decrease their T_b_ to within a normothermic range before engaging in any further flight activity. We thus tested, at both the inter- and intra-individual level, that the duration between flights is governed by the time it takes to become normothermic. Finally, we (3) quantified the rate of body cooling and the time to reach a minimum T_b_, during stops between flights, in order to evaluate if it could influence migration speed.

## Materials and methods

### Model species and population studied

Common eiders are large (2 kg) sea ducks that dive for food living in the benthos. They are characterized by short-pointed wings resulting in high wing-loadings and high flight speed (Day et al., 2004; Guillemette and Ouellet, 2005). The population studied is breeding in Baltic and molting in the Wadden Sea (Rigou and Guillemette, 2010). During migration, eider ducks usually follow the coast and rarely fly over areas of unsuitable feeding habitats (Guillemette, 2001; Guillemette et al., 2016). As with many other species of waterfowl, common eiders undergo a molt migration in late summer. They move from breeding habitats to their molting areas where they lose their wing feathers all at once leading to a period of flightlessness that last 36 days on average (Guillemette et al., 2007). The molt migration of this population in summer, involves movement from the breeding grounds in the central Baltic to the molting quarters located in the Wadden sea, covering a distance of 714 km (±286) on average.

The study was performed on Christiansø Island (55°19′N, 15°12′E), an old Danish fortress located in the southern Baltic Sea, 18 km from the Danish island of Bornholm. The general approach of our work involved the monitoring and deployment of data loggers on breeding females, using heart rate data to determine the start and the end of each flight, and computing variation of body temperature during and between flights. Using the mean eider groundspeed velocities estimated at 83.5 ± 0.3 km h^−1^ by Day et al. (2004) and the total time spent flying (see below) during each migration day of each individual, we calculated the migration distance of each instrumented female. Using that information together with the molting movements of this Danish population, as described previously (Lyngs, 2000), we estimated the location and used the date of migration for all individuals to estimate air [15.5 ± (*SD*) 1.8] and (surface) water temperature [15.5 ± (*SD*) 1.9].

### Deployment of data loggers

On Christiansø island, 45 common eiders were captured in 2003, 2004, and 2005 and implanted with custom made data loggers (DLs, Biometistics, A. J. Woakes) under license from Dyreforsøgtilsynet (Danish Royal Veterinarian Corporation) and approved by the Canadian Council of Animal Care (# CPA 16-03-07-01). Only females were caught as males leave the colony when incubation starts. All surgical procedures were conducted indoors according to the procedure described by Guillemette et al. (2002). The 45 DLs were 36 mm long (±*SD* = 0.5) × 28 mm (0.2) wide × 11 mm thick (0.3) and weighed 21 g (0.3), that is 1.2% of body mass at implantation (Guillemette et al., 2007). Thirty nine (87%) experimental females returned to the study area 1 year later, which is similar to previously reported survival rate in that species (Coulson, 2008). The last result being most likely related to the fact that implanted DLs do not alter aerodynamic and hydrodynamic properties of experimental individuals (Guillemette et al., 2002; White et al., 2013). One year after the implantation, 36 females were re-captured of which 17, 7, and 12 (respectively for 2003, 2004, and 2005) had their data logger removed. For all studied years, data loggers recorded pressure and heart rate every 2 s and body temperature every 16 s, except for 2003 females as the temperature sensor was not operational for that deployment. We thus analyzed for this paper data from 19 females (2004 and 2005 deployment only).

### Time spent flying

Flight schedules (number and duration of flights) were compiled for each bird following the method described by Pelletier et al. (2007). This method is based on the dramatic increases and decreases of heart rate upon take-offs and landings respectively, where heart rate is typically three to four times the resting level. For every female, the daily time spent flying (TSF) was obtained by summing the duration of all flights that occurred during 1 day.

### Body temperature during a flight cycle

**Figure 1:**
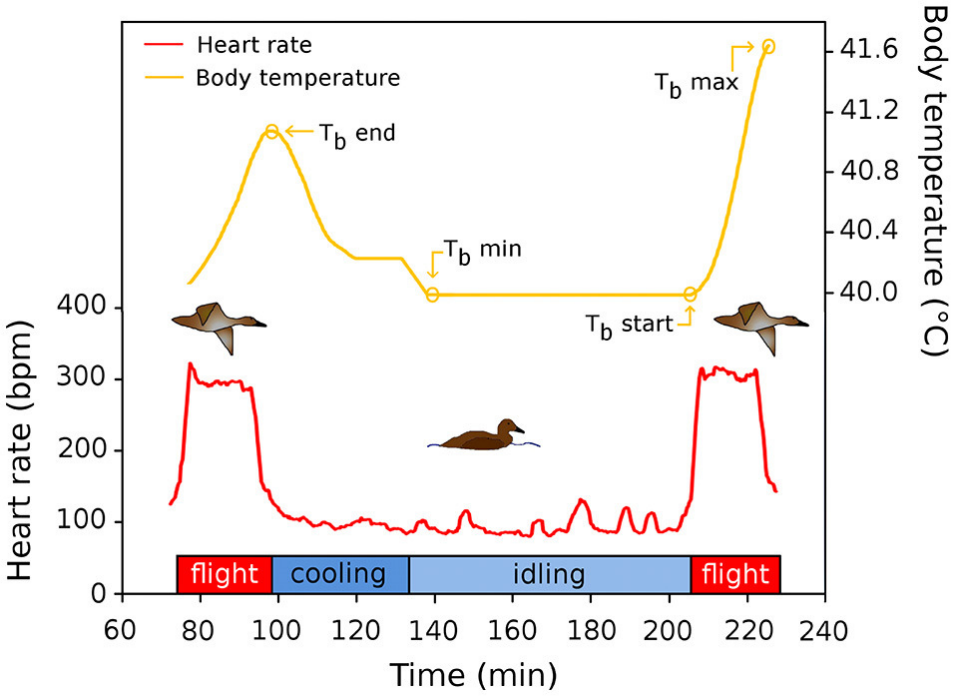
Schematic representation of a flight cycle during migration of common eiders. The red line depicts the heart rate of two flights, and the time interval between flights, used to quantify flight occurrence and flight duration of this study. The yellow line shows the (smoothed) variation of T_b_ in relation to time in the flight cycle. See text for definitions of acronyms.

A flight cycle is composed of flight followed by a period of time spent on the water before the next flight (stop). Body temperature (T_b_) was recorded at the start (T_bStart_) and at the end (T_bEnd_) of each flight together with maximum T_b_ (T_bMax_), whenever it occurred during each flight (Figure 1). The T_b_ sensor time inertia was evaluated to be 3 min as we observed changes in T_b_ only after 3 min of flight. Thus, all flights <3 min were excluded from our analysis (*n* = 412). In addition, the minimum T_b_ (T_bMin_) occurring during each stop interval was recorded. We define the heat storage index (HSI) as T_b_ in relation to elapsed time (°C. h^−1^). Here, we calculate the heat stored during flight for each time interval and hence report the interval specific HSI for each flight segment (0–5, 5–10 min). Similarly, we computed a body cooling index (BCI) by calculating the difference of T_b_ between the end of flight and T_b_ at 5 min increment after the end of each flight.

### Data analysis

First, we were interested to know how and if eiders ducks were able to regulate T_b_ during migration at a daily scale compared to periods before and after migration. Daily body temperature (T_bDaily_) during migration was calculated as the average of all T_b_ datum (5,400 per day) recorded during migration days of each individual. This average value was subtracted in a before-after fashion using a similar time interval (3 days), giving two individual deltas per individual. These deltas were averaged over the 19 females for which confidence intervals were computed using the bootstrap method (see main text for results).

Second, we were interested to know if body cooling between flights was sufficient to dump all the heat gained during flight. We started our analysis by relating the heat gain during flight with heat loss during stops at the intra-individual level. We calculated an intra-individual reduced-major axis (RMA) slope between the temperature gained during all flights performed in relation to the temperature lost between flights (stops) for each individual taken separately and then tested if the intra-individual slope averaged over all individuals differs from the theoretical value of one (i.e., complete T_b_ regulation). The average slope and its associated confidence intervals (CI) was computed using a bootstrap and 10,000 re-samplings (Lunneborg, 2000). In a further step, we tested the complete regulation hypothesis at the inter-individual level by summing all gains and losses in T_b_ (in relation to T_bStart_) occurring during an average migration day for each individual in turn. From these data, a RMA equation was computed where the correlation coefficient was tested for significance using a permutation test.

Finally, we calculated the intra-individual correlation level between T_bStart_, T_bEnd_, T_bMax_ for all flights performed, which was averaged over all individuals with CIs computed from a bootstrap and ten thousand re-samplings.

## Results

**Figure 2:**
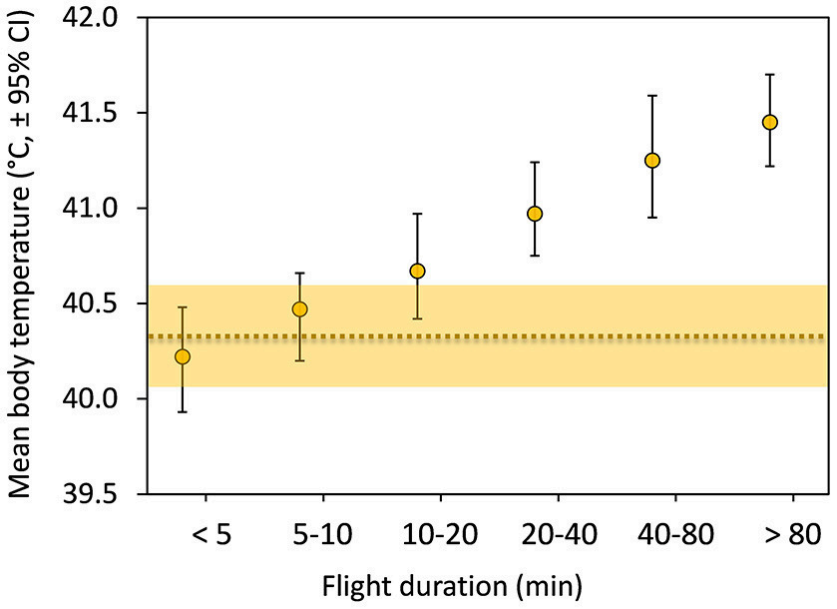
Mean body temperature (T_bMean_) in relation to flight duration with error bars depicting (95%) bootstrap confidence intervals (*N* = 19 females, *n* = 412 flights). The horizontal dotted line represents the daily (24 h) body temperature (T_bDaily_) of the 19 females during a total of 49 days of migration together with (shaded area) 95% bootstrap confidence intervals.

Mean T_b_ during flight increased positively with flight duration during the molt migration of the common eiders, from about 40.2°C for short flights to 41.5°C for longer flights (Figure 2). Mean T_b_ of short flights (3–10 min) were within the mean daily variation of T_b_ (T_bDaily_) during migration days and only longer flights (>10 min) were outside the confidence intervals (Figure 2). Given that migration effort of these 19 females is slightly more than 200 min spent flying on average per day (Guillemette et al., 2016), we tested if average T_b_ at a daily scale would be influenced by flight time.

**Figure 3:**
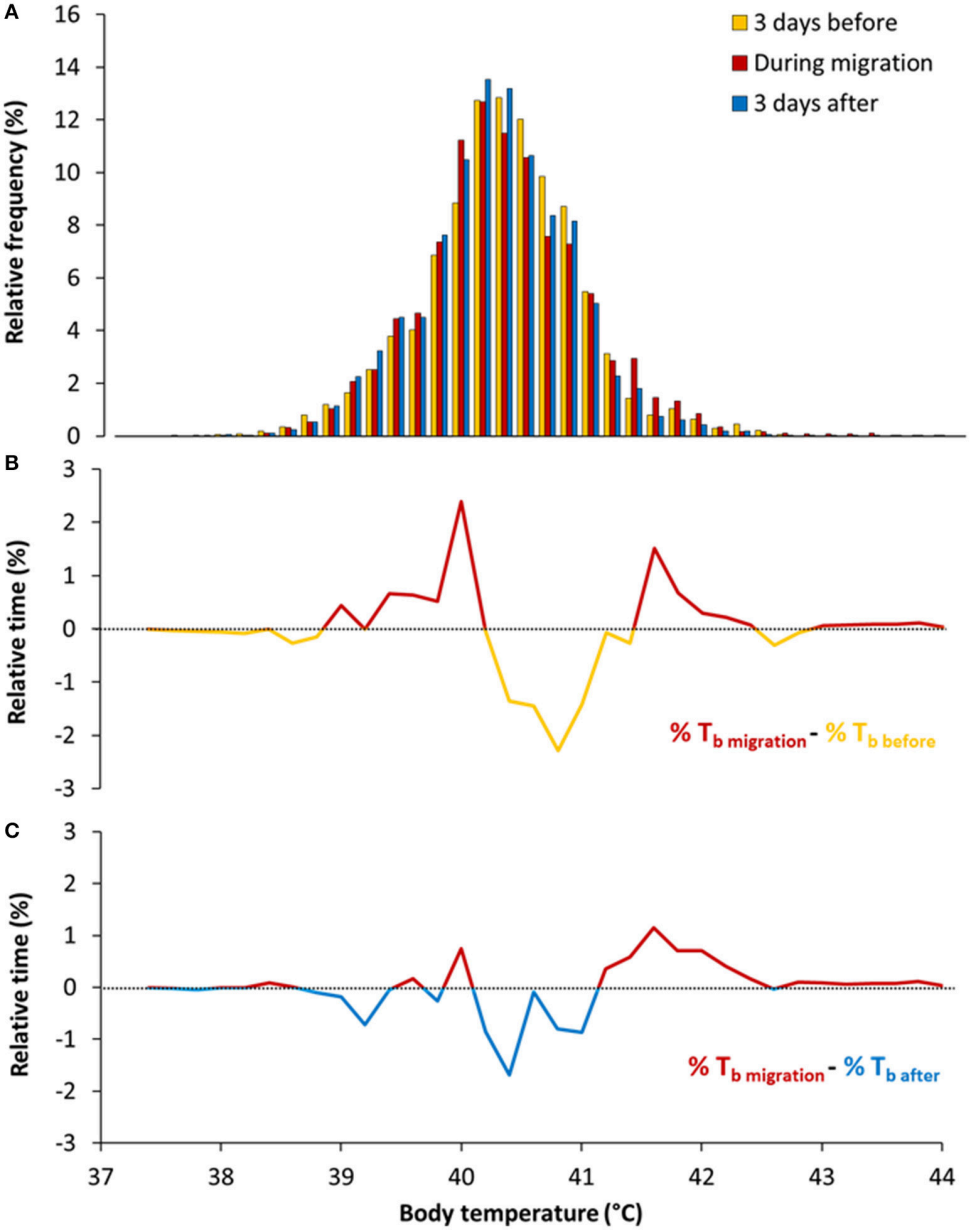
**(A)** Frequency distribution of daily T_b_ during, before and after migration. Given that implanted loggers sampled regularly T_b_ (every 16 s) during each day of migration, the frequency distribution is giving the relative (%) time spent in each T_b_ classes (0.2°C). **(B)** Differences in relative time in T_b_ between migration and 3 days before migration (delta migration-before). **(C)** Differences in relative time in T_b_ between migration and 3 days after migration (delta migration-after).

This was achieved by comparing T_bDaily_ during migration with periods before and after migration (Figure 3). We found that T_bDaily_ before migration [40.41 ± (*SD*) 0.58°C] was slightly higher, on average, than during migration [40.36 ± (*SD*) 0.54°C], as the mean difference between the two temperatures (before and during migration) exclude zero [average difference = 0.05°C ± (bootstrap CI) 0.04]. In contrast, T_bDaily_ after migration [40.30 ± (*SD*) 0.54°C] was lower on average than during migration [average difference = 0.06°C ± (bootstrap CI) 0.06]. Although, T_bDaily_ varied little but significantly, on average, the shape of the frequency distribution was influenced by flight and migration. Unsurprisingly, during migration eiders experienced more T_b_ between 41.4 and 42.2°C when compared to the before and after migration periods (Figures 3B,C).

**Figure 4:**
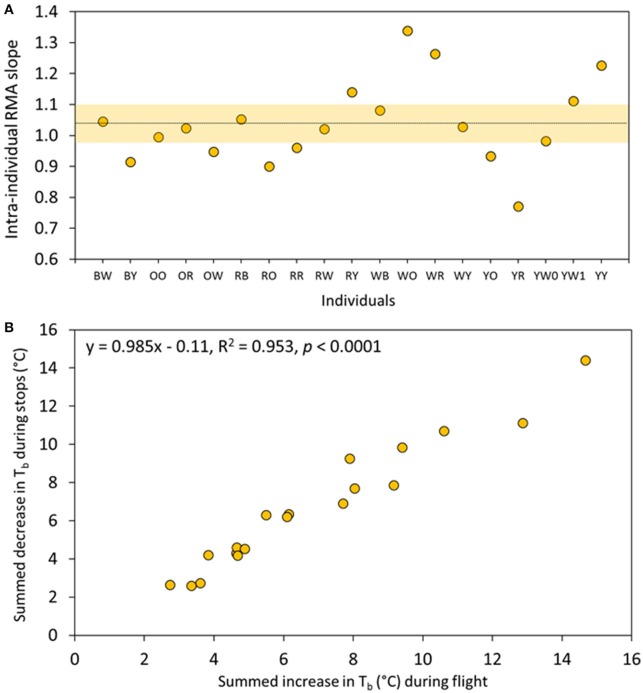
Linear relationships between the numbers of °C gained during flight and the number of °C lost during stops between flights. **(A)** Intra-individual relationships where each point represents a RMA slope for each female eider duck of this study. The dotted line depicts the average slope (1.04) for all individuals while the shaded area shows the (bootstrap) confidence intervals (see Section Materials and Methods). **(B)** Inter-individual relationships where each point is an individual for which the number of °C gained or lost during a flight cycle were summed during an average migration day.

We then tested the idea that body cooling between flights was a determining factor in the migration strategy of eiders, by relating the body heat gain during flight with body heat loss during stops, at the intra-individual level. We calculated an intra-individual RMA slope between the temperature increases during flights in relation to the temperature decreases between flights for each flight cycle taken separately, and then tested if the average intra-individual slope differs from the theoretical value of one (i.e., complete T_b_ regulation). The RMA slope ranged between 0.75 and 1.35 among individuals, with a mean of 1.04 ± 0.06 (bootstrap CI) when averaged over all individuals (mean Pearson *r* = 0.737, Figure 4A). We further tested this hypothesis at the inter-individual level by summing all increases and decreases in T_b_ occurring during an average migration day for each individual in turn; we also found a strong and linear relationship (y = 0.985x–0.11, *r* = 0.976, permutation test, *p* < 0.0001; Figure 4B). Notably, cumulative daily individual variation of T_b_ related to flight activity during migration was high (3–15°C).

**Figure 5:**
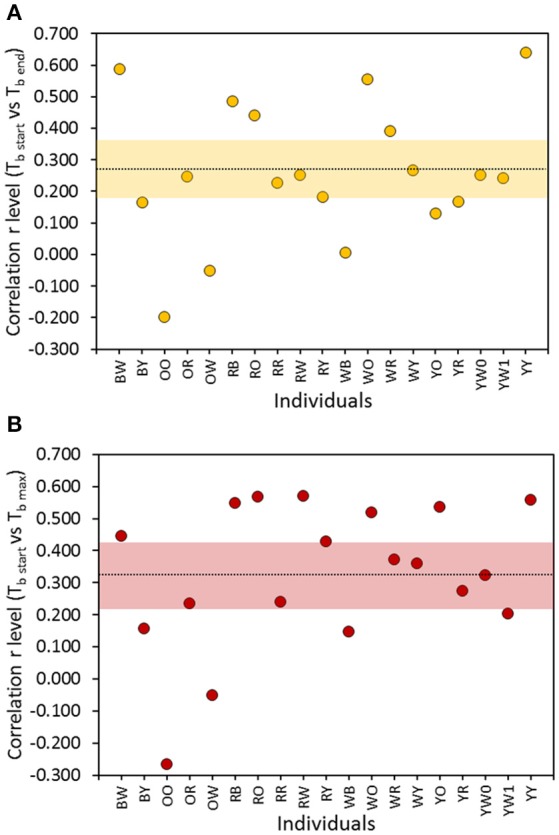
Correlation level between Tb at the start of a flight (T_bStart_) and **(A)** T_b_ at the end (T_bEnd_) of a flight (yellow circles) and **(B)** maximum T_b_ (T_bMax_) occurring while aloft (red circles) at the intra-individual level. The overall average (*n* = 19) correlation level is shown with 95% (bootstrap) confidence intervals (shaded area).

The close regulation of T_b_ observed (Figure 4) at the scale of a flight cycle led us to postulate that reduction of T_b_ to a minimum level is likely to be mandatory, as it would reduce the likelihood of an occurrence of reaching an unacceptable level of hyperthermia for the subsequent flight. Despite the seemingly large variation between individuals, body temperature at the start of a flight (T_bStart_) was positively related to body temperature at the end (T_bEnd_) of a flight at the intra-individual level [average correlation = 0.263 ± (bootstrap CI) 0.095, Figure 5A]. A similar observation was made for T_bMax_ [average correlation = 0.325 ± (bootstrap CI) 0.101] which supports our hypothesis (Figure 5B). In addition, at the inter-individual level (results not shown), a similar relationship with T_bStart_ reveals a significant coefficient of correlation for both T_bEnd_ (0.664, *p* < 0.05) and T_bMax_ (*r* = 0.685, *p* < 0.05).

**Figure 6:**
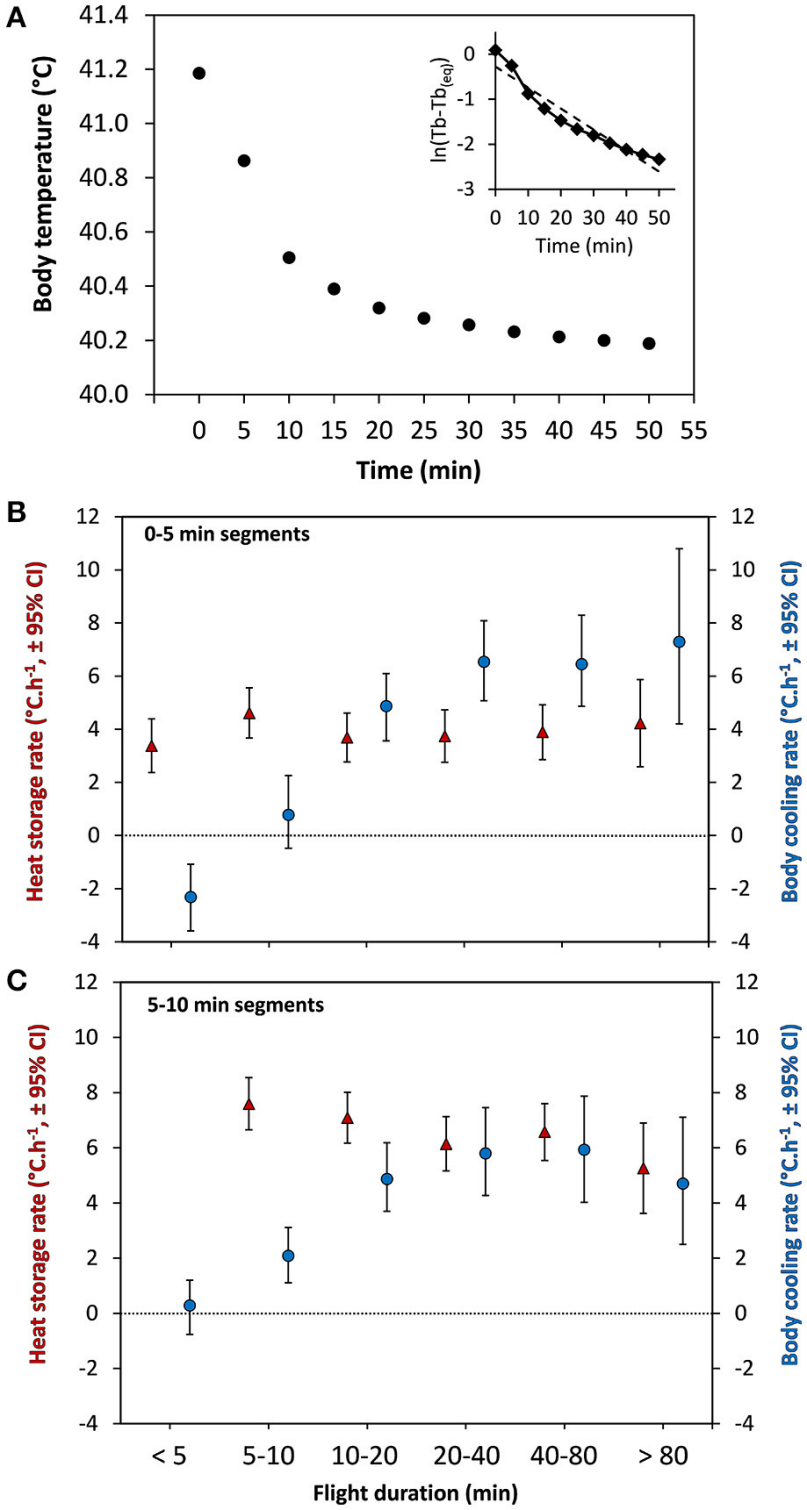
Body temperature and cooling rates in relation to time from the end of a migratory flight. **(A)** Average body temperature (T_b_) in relation to time for all flights performed by the 19 females. The curve follows an exponential decrease with highest body cooling rates for 0–5 (representative of flights <5 min) and 5–10 min cooling segments. (**A**, insert) Natural logarithm of the adjusted T_b_ values (T_b_ minus the equilibrium T_b_) plotted against time (min). The dashed line is the linear regression through these data representative of the relationship expected from a Newtonian cooling curve **(B)** Rate of 0–5 min cooling segments during stops (blue circles) in relation to flight duration compared to heat storage rate during flights (red triangles) for similar independent segments (0–5 min) as computed by Guillemette et al. (2016). **(C)** Rate of 5–10 min cooling segments during stops (blue circles) in relation to flight duration compared to heat storage rate during flights (red triangles) computed by Guillemette et al. (2016). Note that heat storage rate for 5–10 min segments cannot be presented for flight duration of 0–5 min.

During temporary migratory stop-overs, the body temperature of the eiders decreased at an exponential rate, which stabilized at about 45–50 min, on average, from the end of the preceding flight (Figure 6A). Indeed, the highest average cooling rate was during the first 10 min after landing, which itself was substantially higher following longer flights, reaching 6–7°C. h^−1^ (Figures 6B,C). Nevertheless, the average time required to cool down to T_bMin_ [40.10 ± (*SD*) 0.56], the minimum level between flights [47.14 ± (*SD*) 21.73 min], is substantially shorter than the average duration of each stop between flights [72.1 ± (*SD*) 21.6 min], which raises the question of why birds are taking extra time during stops before resuming flight.

## Discussion

In a former paper, we have presented evidence that hyperthermia might dictate flight duration in a migrating bird (Guillemette et al., 2016). Here, we present evidence that body cooling is an integral part of the migration of common eiders by showing that daily fluctuations in T_bs_ caused by flight were precisely regulated at the scale of a flight cycle, despite large cumulative deviations in T_b_ caused by the migration and associated flights. We thus suggest below that such a level of regulation results from behavioral thermoregulation together with the intrinsic physiological flexibility of birds.

### T_bDaily_ and heat dumping during stop-overs

It is apparent that birds have higher body temperature tolerance in comparison to mammals (Prinzinger et al., 1991; McNab, 2002). During molt migration, Baltic common eiders fly 205 min per day, on average (Guillemette et al., 2016), and individuals experience daily cumulative variations in T_b_ ranging between 3 and 15°C (Figure 3B). Despite this large variation in T_b_ associated with flight activity, T_bDaily_ during migration days (40.36 ± 0.54°C) was only slightly different, by 0.05 degrees on average, when compared to before (40.41 ± 0.58°C) and after (40.30 ± 0.54°C) periods. The most distinctive features of migration were the time spent at T_b_ values in the range of 41.4–42.4 and 40.0–40.2°C (Figures 2B,C), which correspond respectively to T_b_ encountered during long flights (see Guillemette et al., 2016, Figure 1) and T_b_ while idling during stops. The observation that eiders regulate T_b_ around T_bMin_ at 40.1°C during stops, idling between flights, suggests a selected body temperature target while waiting to perform the next flight. It is also interesting to note this temperature range to be at the low range of T_bDaily_ confidence intervals (Figure 2) together with the fact that average T_b_ during flights of 0–10 min are within the confidence intervals of T_bDaily_. Altogether, it thus appears that migrating common eiders defend T_bDaily_ with success and we suggest that a part of the absolute level of hyperthermia reached by an exercising animal, the time spent at a high level of T_b_ is also an important point to consider when evaluating the potential and deleterious effects of hyperthermia. Indeed, the return to normothermia was relatively rapid in migrating eiders (Figure 6A). Support for this interpretation is given by various cancer treatment therapies using hyperthermia in humans (Wang et al., 2013), which induces intracellular reactive oxygen species (ROS) production and mitochondrial ROS generation in a time-dependent manner. One advantage of maintaining T_b_ at a low level before the next flight is to eventually reduce the level of hyperthermia reached during that subsequent flight. This hypothesis was supported by the data as we found a positive correlation between T_bStart_ and T_bEnd_ and T_bStart_ and T_bMax_ (see Section Results).

### Body cooling rate and idling time

It is expected that marine birds potentially dissipate heat at a high rate following landing, due to being on water. Indeed, the rate of cooling of migrating common eiders is high for the first 10 min after landing, particularly after longer flights (Figures 6B,C), with average rates of cooling ranging from 6 to 7°C. h^−1^. These estimates of body cooling are difficult to compare with any other species, given the paucity of data. From birds shot while flying, Platania et al. (1986) observed a decrease of T_b_ varying between 1.2 and 3.6°C h^−1^ for three species (222–449 g), 9.0°C h^−1^ for the smallest species (40 g) and 1.7°C h^−1^ for the largest species (765 g). Unfortunately, Platania et al. (1986) do not give any details about the medium (air vs. water) where these shot birds were cooling down when taking the measurements. One further complication is that we expect dead birds to cool down more slowly than live birds given they cannot divert blood flow to the periphery or legs to cool down more rapidly (see below).

Interestingly, the cooling rates observed during stop-overs of eiders are similar to the highest heat storage rates occurring at the beginning of a flights (Figures 6B,C), indicating that body cooling and heat storage reach equivalent maximum values. In his review, McNab (2002) also reports similar values for ectotherms for both cooling and heat storage rates in air. However, it could be expected that body cooling in aquatic birds would occur at faster rates than heat storage, given the heat dissipation potential of sea water is much higher than air. In humans, cooling rates have been measured in circulated water baths (14 and 20°C water) after exercise in air (65% VO_2Max_ at 38°C) and reaching a rectal temperature of 40.0°C (Proulx et al., 2003). They observed an exponential decrease of T_b_ with the largest decrease occurring in the first 10 min after immersion, similar to eiders during stop-overs (Figure 6A), with the difference that maximum (11.4–15.6°C h^−1^) and average (9–11.4°C h^−1^) cooling rates were much higher than average heat storage rates (3–4°C h^−1^) in the seven human subjects. Unfortunately, Proulx et al. (2003) did not report the maximum rate of heat storage though a maximum rate of 6°C h^−1^ was reported in a very similar experimental setting (González-Alonso et al., 1999). Thus, although humans are much larger animals, maximum body cooling in water seems to be much more rapid than maximum body cooling of migrating eiders, the other main difference being, of course, that humans are naked and deprived of fur or body feathers.

The naked legs of many bird species (Steen and Steen, 1965; Kilgore and Schmidt-Nielsen, 1975; Midtgård, 2008) have been shown to serve as controlled heat conduits, being of paramount importance for thermoregulation in both water (Steen and Steen, 1965; Kilgore and Schmidt-Nielsen, 1975; Midtgård, 2008) and air (Baudinette et al., 1976; Martineau and Larochelle, 1988). The evidence for this is stemming from calorific studies and blood flow measurements. For example, blood flow to the legs of mallards (*Anas platyrhynchos)* and fulmars (*Macronectes giganteus)* is reduced and heat conservation occurs (Johansen and Wesley Millard, 1973; Kilgore and Schmidt-Nielsen, 1975), when water temperature is reduced experimentally during rest. In contrast, heat dissipation can increase considerably where legs are serving as heat dumping organs during both flying and swimming, although the rate of heat loss can vary considerably given the varying experimental conditions used by the different studies. From a behavioral point of view, trailing or retracting the legs and feet in the plumage while flying (Udvardy, 1983; Neumann and Neumann, 2016) or swimming (Harris et al., 2010) is further modulating heat dissipation of birds.

### Time as the cost of behavioral thermoregulation

The fact that eiders stop flying when overheating can be seen as another example of behavioral thermoregulation. In his thorough review, McNab (2002) concluded that time is the major cost associated with behavioral thermoregulation of ectotherms although his book almost eludes the subject in relation to endotherms, most probably because examples were few (Smit et al., 2016). Here, we propose that cooling time is a major cost of the migration strategy of common eiders. As such, eiders take about 47 min on average to cool down by 1.1°C and reach T_bMin_, corresponding to an overall cooling rate of 1.4°C h^−1^, which is much lower than the max rate observed at the beginning of the cooling period (6–7°C h^−1^, Figure 6). This is readily explained by the (exponential) nature of the cooling curve and the fact that most flights of eiders during molt migration are short (16 min average, Guillemette et al., 2016) when the level of heat stored in the body is still relatively low (Figure 2). Interestingly, body cooling in eiders does not appear to be a passive cooling process as predicted by Newton's law of cooling because the relationship between ln(T_b_-T_b(eq)_) and time (see Figure 6A insert), does not fit a linear relationship. In fact, it appears that cooling is initially enhanced and slows down progressively just before reaching the level of equilibrium. Although speculative, the initially increased rate of cooling may be indicative of an active processes of heat dissipation related to e.g., an increased blood flow through the legs of the birds in cooler water.

However, it remains that the 47 min to cool down is still lower than the average duration of stops between flights observed in our sample (72.1 ± 21, 6 min). This indicates that the time spent between flights is not entirely devoted to body cooling (Figure 1) and we hypothesized that such an extra time taken between flights is caused by unfavorable wind conditions. Nevertheless, assuming that cooling down to T_bMin_ is a prerequisite to perform the next flight, the daily time spent cooling down on a typical migration day (with about 14 flights per day, Guillemette et al., 2016) would represent 523 min or 36% of the 24 h of the daily time budget. If we add the idling time (about 300 min or 20%) we obtain that eiders are spending altogether 56% of the time spent cooling and idling, between flights, during a typical migration day. This indicates that behavioral thermoregulation of common eiders during migration represents a major component of their time budget.

In conclusion, it is likely that the occurrence of hyperthermia in common eiders is due to their high wing loading and costs of flight, which in turn is a consequence of their morphology being adapted for diving. A fruitful future research direction would be to establish the prevalence of hyperthermia in other diving bird species during migratory flights, and whether some species show flight altitudinal changes in direct response to increases in T_b_. It is likely hyperthermia is a hitherto underestimated contributing factor to migration strategies, and the possible daily distances that can be traveled. Moreover, hyperthermia may be one of major contributing factors of why other diving species with high loading (e.g., alcids) undertake a significant portion of their migrations swimming as opposed to flying (Olsson et al., 1999), with the constant contact of their feet with the water, coupled with the lower work-rate of the leg muscles compared to flight, ameliorating the effects of hyperthermia.

## Author contribution

MG, DP conceived the ideas, conducted the experiments, prepared the manuscript and figures, and approved the manuscript. EP and SP prepared manuscript, assisted in analysis and approved the manuscript.

### Conflict of interest statement

The authors declare that the research was conducted in the absence of any commercial or financial relationships that could be construed as a potential conflict of interest.
